# Evidence that catecholaminergic systems mediate dynamic colour change during explosive breeding events in toads

**DOI:** 10.1098/rsbl.2022.0337

**Published:** 2022-10-19

**Authors:** Susanne Stückler, Matthew J. Fuxjager, Doris Preininger

**Affiliations:** ^1^ Department of Cognitive Biology, University of Vienna, Austria; ^2^ Department of Ecology, Evolution and Organismal Biology, Brown University, Providence, RI, USA; ^3^ Department of Evolutionary Biology, University of Vienna, Austria; ^4^ Vienna Zoo, 1130 Vienna, Austria

**Keywords:** Asian common toad, *Duttaphrynus melanostictus*, dynamic dichromatism‌, epinephrine, norepinephrine, vision model

## Abstract

Many animals communicate by rapidly (within minutes or seconds) changing their body coloration; however, we know little about the physiology of this behaviour. Here we study how catecholaminergic hormones regulate rapid colour change in explosive breeding toads (*Duttaphrynus melanostictus*), where large groups of males gather and quickly change their colour from brown to bright yellow during reproduction. We find that both epinephrine (EP) and/or norepinephrine (NE) cause the toads' skin to become yellow in minutes, even in the absence of social and environmental cues associated with explosive breeding. We hypothesize that natural selection drives the evolution of rapid colour change by co-opting the functional effects of catecholaminergic action. If so, then hormones involved in ‘fight or flight’ responses may mechanistically facilitate the emergence of dynamic visual signals that mediate communication in a sexual context.

## 1. Introduction

Many species rapidly and reversibly change their body colour [1–4]. Iconic examples include cephalopods and chameleons [4], which alter their colours within seconds to provide instant camouflage, signal conspecifics [5], court mates [6] or display aggressive motivation [7]. Yet, functions of rapid colour change are often better studied than the physiological mechanisms that support it [8]. While past work implicates a variety of hormonal and/or neuronal systems that help mediate quick changes in body coloration patterns [1], the precise nature of these systems remains unclear.

In at least 178 anurans, males use rapid colour change (dynamic dichromatism) to mediate socio-sexual interactions during the breeding season [9]. This behaviour is especially important for species that engage in explosive breeding, where all sexually receptive individuals arrive synchronously at a spawning site, and breeding takes place over a period of a few days. Explosive breeding events are characterized by male-biased sex ratios, with intense competition among males, who then scramble to access female mates. Males consequently experience low rates of reproductive success, whereas females experience relatively high rates of mortality [10]. When dynamic colour change preceeds explosive breeding, it therefore likely regulates sexual recognition by helping males more easily distinguish each other from females [11–13]. In this way, we might consider sexual dichromatism that occurs prior to explosive breeding events an adaptive strategy that facilitates the speedy acquisition (and subsequent loss) of a signal for intense male–male competition [9]. If this is true, however, then mechanisms must evolve to facilitate colour change in anticipation of reproduction.

For most species, sudden and dramatic changes to the environment trigger explosive breeding, including the onset of monsoon rains and fluctuations in temperature, humidity, or barometric pressure [14]. These environmental perturbations—particularly when paired with increases in social stress—can engage animal stress systems [15–17], including the highly conserved stress systems that mediate adaptive ‘fight or flight’ responses [18]. This latter system involves the release of the catecholamine hormones epinephrine (EP) and norepinephrine (NE) [19], which both act to modify an organism's physiology to better contend with stressors. Importantly, catecholamines also influence skin colour, as these hormones' receptors are present on most chromatophores [20]. Such effects can occur within minutes of catecholamine release [21,22], resulting in either skin lighting [23,24] or darkening [25,26]. We hypothesize that natural selection drives the evolution of rapid colour change during explosive breeding events by co-opting the functional effects of catecholaminergic hormone action, which originally arose to mediate stress responsivity. We, therefore, expect that (1) colour change is a by-product or direct effect of a stress response triggered by cues associated with the onset of explosive breeding events, and (2) stress-related EP and/or NE pathways help activate rapid colour change even in the absence of social stimulation (which might be required later on to maintain colour change). We examine this idea in Asian common toads (*Duttaphrynus melanostictus*), where males change from an inconspicuous brown (similar to females) to bright yellow immediately prior to the formation of explosive breeding aggregations. This behaviour is triggered by monsoon rains, and causes greater than 200 individuals to arrive synchronously at spawning sites during the daytime. We, therefore, test for the effects of EP and NE on toad body coloration over a 12-h period at the Vienna Zoo, as well as whether these colour changes are sufficient for receiver discrimination.

## 2. Methods

The Vienna Zoo houses semi-free ranging Asian common toads in the rainforest house (greater than 1000 m^2^). We caught 25 males from this population and placed them in four terraria (100 × 50 × 50 cm) equipped with hiding structures, water bowls, and humid and dry zones (mean temperature: 25 ± 1°C; mean humidity 74 ± 17%). Toads were housed under a 12-h light/dark cycle, and were fed with vitaminized crickets every second day.

On three separate experimental sessions, we examined the effect of EP and NE treatment on skin coloration (from December 2020 to February 2021). In each session, we randomly assigned 15 males to three different treatment groups (EP, NE or control; *n* = 5 per group). Body size (mean/s.d. = 49.05 ± 0.83 mm, range = 40.19–58.42 mm, *n* = 25) and weight (mean/s.d. = 17.31 ± 0.91 g, range = 11.17–29.05 g, *n* = 25) were similar among each treatment group of each session (Kruskal–Wallis: size: *K* = 14.351, *p* = 0.0731; weight: *K* = 10.402, *p* = 0.238), suggesting similar age. We allowed four weeks to pass between sessions, as this time exceeds an adequate period that ensures all catecholamines have cleared from the toads’ circulation [27–29].

Each experimental session consisted of two parts: (1) priming with human chorionic gonadotropin (hCG) and (2) treatment with different catecholamines or saline solution (control). Before part 1, we measured the toads' size (SVL, mm), weight (g) and back reflectance (baseline 1). Subsequently, we primed each toad with a single injection of 30 µl hCG (Sigma-Aldrich CG10-1VL; 20 IU g^−1^ bodyweight in saline solution) and placed them in their respective fauna box (20 × 19 × 20 cm) for a period of 5 h. hCG is a common exogenous reproductive hormone, widely used to induce reproduction and breeding behaviour in amphibians [30,31]. To verify the impact of hCG injections on body coloration, we visually checked the individuals body coloration hourly. Pigmentation changes between the toad's general brown and the conspicuous nuptial bright yellow coloration are highly noticeable to human observers and easy to assess. After 5 h, we again performed reflectance measurements of all individuals (baseline 2) followed by the respective EP, NE or saline treatments via 50 µl intraperitoneal injection in the dorsal lymph sac. The test groups were administrated a dose of (1) 50 µl EP (Sigma-Aldrich E4250; 1.8 µg g^−1^ bodyweight), (2) 50 µl NE (Sigma-Aldrich A7256; 0.4 µg g^−1^ bodyweight), or (3) 50 µl saline (0.9% NaCl) as a control. Drug dosages were chosen according to the weight of the species based on prior studies in fish and amphibians [21,22,32,33]. In all experiments, we minimized handling time to less than 5 min to reduce unintended stress effects on circulating levels of catecholamines.

Back coloration was measured 30, 60, 120, 180 min and 12 h after treatment (electronic supplementary material, figure S1) with a spectrometer (JAZ series; Ocean Optics, Dunedin, USA) according a standardized protocol. We used Avicol v6 software [34] to extract brightness, hue and chroma measures—the essential parameters to describe coloration from reflectance spectra (see electronic supplementary material) [35].

Significant effects of our treatments on the colour variables do not necessarily imply that these effects can be perceived by conspecific rivals or mating partners. Thus, we additionally quantified whether spectral differences could be detectable by toads using a colour vision model with the package ‘pavo’ [36]. We thereby examined the ability of toads to discriminate among untreated females (*n* = 15), control group males, EP and NE treated males, 30 min after the treatment (see electronic supplementary material).

Statistical analyses were performed with the program R (v. 3.4.2; R Studio Team 2021) and SPSS 26 (IBM SPSS Statistics, USA). To test the impact of hCG on body coloration, we compared colour parameters of baseline 1 and 2 using generalized linear mixed models (GLMMs) with normal distribution, identity link function and Student's *t* statistic with sequential Bonferroni correction for *post-hoc* tests. The colour parameters were entered as dependent variables, with measurement point (baseline 1 or 2) as predictor variables and the identities of male and session as random variables to correct for repeated measurements. Next, we visually determined colour change from brown to yellow (yes/no) and used Fisher's exact test (with sequential Bonferroni correction) to test for differences in the odds individuals change colour among treatment groups. To understand what colour parameters (brightness, hue or chroma) determine yellow coloration, we selected measurements of males that were affected by respective hormone treatments (EP, NE) and compared them to the control with GLMMs. The colour parameters were entered as dependent variables, with treatment, measurement point (30, 60, 90, 120 min, 12 h) and treatment × measurement point as predictor variables and male and session as random variables. Finally, to test conspecific discriminability we performed a GLMM on just-noticeable differences (JNDs) 30 min after treatment. Chromatic and achromatic differences were entered as dependent variables, treatment as predictor variable and identities of males and females as random variables.

## 3. Results

**Figure 1.**
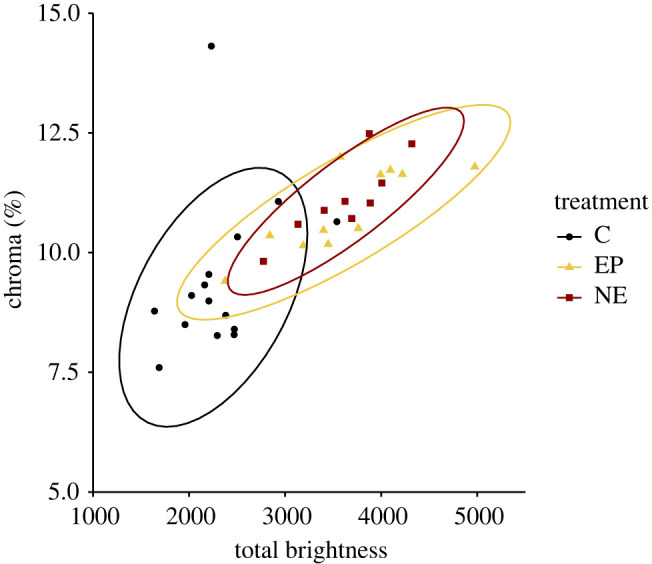
Back coloration of males 30 min after EP/NE and control treatment. Compared are colour parameters of the control (*n* = 15), EP (*n* = 11) and NE (*n* = 9). Symbols denote individual data points, while ellipsoid boundaries represent mean ± 95% confidence interval of colour parameters.

Priming males with hCG had no effect on coloration (GLMM (B1 versus B2): brightness: *F*_1,88_ = 1.084, *p* = 0.301; hue: *F*_1,88_ = 0.512, *p* = 0.476; chroma: *F*_1,88_ = 0.665, *p* = 0.417; electronic supplementary material, figure S2). By contrast, catecholamine administration triggered significant changes in colour (Fisher's exact test: *p* < 0.001), with the most visible changes occurring 1.5–2 cm around the injection site. Compared to controls, toads receiving EP and NE were brighter yellow (GLMM: *F*_2,160_ = 20.428, *p* < 0.001) and had higher yellow saturation (GLMM: *F*_2,160_ = 34.802, *p* < 0.001; figure 1, electronic supplementary material, table S1). EP/NE had no effect on hue (GLMM: *F*_2,160_ = 1.552, *p* = 0.215; electronic supplementary material, figure S3, S4). Notably, this colour change is similar to that which we see in wild populations of Asian common toads, as individuals migrate to breeding sites.

**Figure 2.**
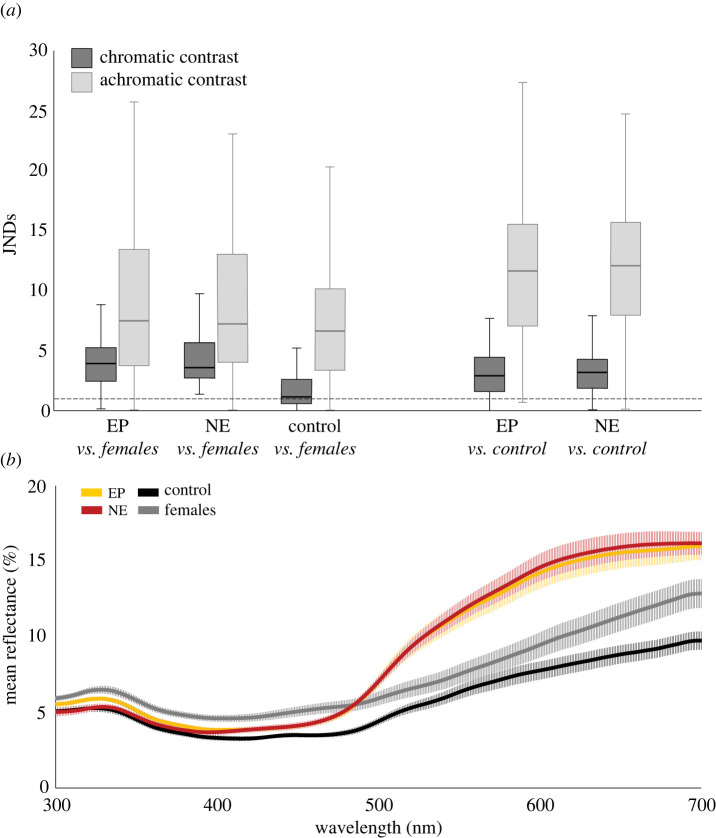
Colour differences of male (30-min post-treatment) and female toads. (*a*) Chromatic and achromatic contrast (expressed as just-noticeable differences (JNDs) of males [control, epinephrine (EP) and norepinephrine groups (NE)] against female body coloration, and contrast of males (EP and NE group) against control group coloration, as perceived by toads (spectral sensitivity of *Bufo bufo*). Dashed line = minimum threshold of discrimination (JND = 1). (*b*) Mean spectral reflectance (including standard error) of back coloration of male (control: *n* = 15; EP: *n* = 11; NE: *n* = 9) and female toads (*n* = 15).

We next assessed contrast among females, brown control males, and hormone-treated yellow males, as viewed by conspecifics (figure 2). Dynamic yellow coloration shows high discernibility in contrast to brown control males (*F*_2-521_ = 7.895, *p* < 0.001; EP versus control: *p* = 0.003; NE versus control: *p* < 0.001). When looking at JND values (GLMM estimated means ± SE), yellow provides sufficient contrast for discrimination between brown control males (EP: dS = 3.022 ± 0.621; dL = 11.552 ± 1.965; NE: dS = 3.328 ± 0.622; dL = 11.998 ± 1.967) and females (EP: dS = 3.768 ± 0.494; dL = 8.833 ± 1.474; NE: dS = 4.196 ± 0.494; dL = 8.812 ± 1.477) in chromatic and achromatic conditions.

## 4. Discussion

Using Asian common toads, we show that catecholaminergic hormones EP and NE help regulate rapid colour change. Reproductively primed males given either hormone increase their skin brightness and yellow colour saturation for at least 1 h, whereas reproductive priming itself (via hCG) has no such effect. This indicates that catecholamine action is sufficient to trigger changes in skin colour from dull brown to bright yellow, which likely mediates socio-sexual communication during explosive breeding events [11,37].

Work in other anuran species similarly shows that catecholamines induce rapid colour change [21,23–26]. However, these studies show that such changes occur *after* individuals engage in sociosexual interactions (e.g. amplexus), suggesting that conspecific encounters are important for hormonal effects on colour to manifest. Our results indicate that this is not the case in Asian common toads; rather, EP and NE change male colour in the absence of any sociosexual interaction. The difference in these results is critical because it implies that stimuli unrelated to male–male and/or male–female conspecific encounters can start off the process of rapid colour change. Our results, therefore, point to the idea that sexual selection exploited (or co-opted) systems that govern adaptive catecholaminergic responses to environmental changes [38–40] to help facilitate evolution of rapid colour change as a display phenomenon. Such responses may have stemmed from abiotic stressors associated with the onset of explosive breeding, such as monsoon rains and/or sudden drops in barometric pressure. Factors, like social stimuli from male–male encounters, are also likely involved (see below). Our captive samples provide first directions, but further experiments—in free-living toads—are needed to more thoroughly pinpoint precise combinations of factors that elicit dynamic colour and underlie its evolution.

Equally importantly is that our results suggest that conspecifics can perceive EP- and NE-mediated changes to toad skin colour. This conclusion comes from results showing that colour difference between treated (yellow) and control (brown) animals are sufficient for conspecific discrimination. Indeed, toads visual systems are capable of colour discrimination through trichromatic colour vision [41], even under low light conditions [42]. Work will be needed to verify this finding in field settings to better establish how individuals navigate complex breeding environments.

Colour change in *D. melanostictus* works exclusively via large melanophores in the sub-epidermis, as individuals have no other chromatophores (xanthophores, erythrophores) [43]. In other anurans, dispersion of yellow pigments in xanthophores and aggregation of dark pigments in melanophores underlies yellow and orange colour [21,22,44]. But without xanthophores, yellow coloration in our toads can only be produced by pigment movement in melanophores. Thus, catecholamines potentially mediate colour change by promoting the aggregation of melanin pigments [8]. This idea is consistent with our findings that EP and NE have no effects on hue, since yellow and brown are virtually the same in this regard [45]. We should, however, acknowledge that our treated individuals only displayed colour changes around the injection site, and some individuals did not change body coloration. As indicated above, this suggests that other factors are likely involved in mediating rapid colour change in our study species; for instance, endocrine regulators such as corticosterone [46], melanophore-stimulating hormone [47,48], and androgenic steroids [44,49] may play a role. This latter hormone is especially intriguing, given that it underlies ‘male-typical’ reproductive behaviour [50] and influence colour in several anurans [44,49]. Similarly, physical and social cues associated with explosive breeding events may interact with catecholamine action to induce a more complete change in male colour.

In sum, our results show that EP and NE help mediate the production of bright yellow coloration in Asian common toads. This finding supports the idea that selection can co-opt catecholaminergic hormone systems to help individuals quickly adopt a discernible colour to optimize behavioural interactions. Mechanisms of stress responsivity may therefore be exploited by evolutionary forces for effective communication in reproductive situations characterized by intense male–male competition for mates.

## Data Availability

The dataset is available from the Dryad Digital Repository: https://doi.org/10.5061/dryad.dbrv15f4b [51]. The data are provided in the electronic supplementary material [52].

## References

[1] Aspengren S, Hedberg D, Sköld HN, Wallin M. 2008 New insights into melanosome transport in vertebrate pigment cells. Int. Rev. Cell Mol. Biol. **272**, 245-302. (10.1016/s1937-6448(08)01606-7) 19121820

[2] Aspengren S, Sköld HN, Wallin M. 2009 Different strategies for color change. Cell. Mol. Life Sci. **66**, 187-191. (10.1007/s00018-008-8541-0) 19112553 PMC11131536

[3] Sköld NH, Aspengren S, Wallin M. 2013 Rapid color change in fish and amphibians—function, regulation, and emerging applications. Pigment Cell Melanoma Res. **26**, 29-38. (10.1111/pcmr.12040) 23082932

[4] Figon F, Casas J. 2018 Morphological and physiological colour changes in the animal kingdom. In eLS, pp. 1-11. New York, NY: John Wiley & Sons, Ltd. (10.1002/9780470015902.a0028065)

[5] Mäthger LM, Hanlon RT. 2006 Anatomical basis for camouflaged polarized light communication in squid. Biol. Lett. **2**, 494-496. (10.1098/rsbl.2006.0542) 17148271 PMC1834008

[6] Herrel A, Dollion A, Marquis O, Leroux-Coyau M, Meylan S. 2020 The colour of success: does female mate choice rely on male colour change in the chameleon *Furcifer pardalis*? J. Exp. Biol. **223**, jeb224550. 32843362 10.1242/jeb.224550

[7] Ligon RA, McGraw KJ. 2018 A chorus of color: hierarchical and graded information content of rapid color change signals in chameleons. Behav. Ecol. **29**, 1075-1087. (10.1093/beheco/ary076)

[8] Nicolaï MPJ, D'Alba L, Goldenberg J, Gansemans Y, Van Nieuwerburgh F, Clusella-Trullas S, Shawkey MD. 2021 Untangling the structural and molecular mechanisms underlying colour and rapid colour change in a lizard, *Agama atra*. Mol. Ecol. **30**, 2262-2284. (10.1111/mec.15901) 33772941

[9] Bell RC, Webster GN, Whiting MJ. 2017 Breeding biology and the evolution of dynamic sexual dichromatism in frogs. J. Evol. Biol. **30**, 2104-2115. (10.1111/jeb.13170) 28833835

[10] Wells KD. 2007 The ecology and behavior of amphibians. Chicago, IL: University of Chicago Press.

[11] Sztatecsny M, Preininger D, Freudmann A, Loretto M-C, Maier F, Hödl W. 2012 Don't get the blues: conspicuous nuptial colouration of male moor frogs (*Rana arvalis*) supports visual mate recognition during scramble competition in large breeding aggregations. Behav. Ecol. Sociobiol. **66**, 1-7. (10.1007/s00265-012-1412-6) PMC3496481 23162205

[12] Gardner KM, Mennill DJ, Savi LM, Shangi NE, Doucet SM. 2021 Sexual selection in a tropical toad: do female toads choose brighter males in a species with rapid colour change? Ethology **127**, 475-483. (10.1111/eth.13156)

[13] Doucet SM, Mennill DJ. 2009 Dynamic sexual dichromatism in an explosively breeding Neotropical toad. Biol. Lett. **6**, 63-66. (10.1098/rsbl.2009.0604) 19793736 PMC2817257

[14] Wilson JX, Sawai H, Kikuchi M, Kubokawa K, Ishii S. 1995 Circulating catecholamine and glucose concentrations in Japanese toads (*Bufo japonicus*) during the breeding season. Gen. Comp. Endocrinol. **98**, 303-310. (10.1006/gcen.1995.1072) 7628689

[15] Carr JA. 2011 Stress and reproduction in amphibians. Horm. Reprod. Vertebr. **2**, 99-116. (10.1016/b978-0-12-374931-4.10006-9)

[16] Adkins-Regan E. 2005 Hormones and animal social behavior. Princeton, NJ: Princeton University Press.

[17] Wingfield JC. 2013 Ecological processes and the ecology of stress: the impacts of abiotic environmental factors. Funct. Ecol. **27**, 37-44. (10.1111/1365-2435.12039)

[18] Lowry CA, Moore FL. 2006 Regulation of behavioral responses by corticotropin-releasing factor. Gen. Comp. Endocrinol. **146**, 19-27. (10.1016/j.ygcen.2005.12.006) 16426606

[19] Romero LM, Gormally BMG. 2019 How truly conserved is the ‘well-conserved’ vertebrate stress response? Integr. Comp. Biol. **59**, 273-281. (10.1093/icb/icz011) 30907954

[20] Maeno N, Iga T. 1992 Adrenergic mechanisms associated with the movement of platelets in iridophores from the freshwater goby *Odontobutis obscura*. Comp. Biochem. Physiol. C **102**, 233-237. (10.1016/0742-8413(92)90106-H) 1358536

[21] Kindermann C, Hero J-M. 2016 Rapid dynamic colour change is an intrasexual signal in a lek breeding frog (*Litoria wilcoxii*). Behav. Ecol. Sociobiol. **70**, 1995-2003. (10.1007/s00265-016-2220-1)

[22] Kindermann C, Narayan EJ, Hero J-M. 2014 The neuro-hormonal control of rapid dynamic skin colour change in an amphibian during amplexus. PLoS ONE **9**, e114120. (10.1371/journal.pone.0114120) 25470775 PMC4254939

[23] Wright MR, Lerner AB. 1960 On the movement of pigment granules in frog melanocytes. Endocrinology **66**, 599-609. (10.1210/endo-66-4-599) 13846098

[24] Abe K, Robison GA, Liddle GW, Butcher RW, Nicholson WE, Baird C. 1969 Role of cyclic AMP in mediating the effects of MSH, norepinephrine, and melatonin on frog skin color. Endocrinology **85**, 674-682. (10.1210/endo-85-4-674) 4308478

[25] Goldman JM, Hadley ME. 1969 The beta adrenergic receptor and cyclic 3′,5′-adenosine monophosphate: possible roles in the regulation of melanophores responses of spadefoot toad *Scaphiopus couchi*. Gen. Comp. Endocrinol. **13**, 151-163. (10.1016/0016-6480(69)90232-9) 4389916

[26] Burgers ACJ, Boschman TAC, Van de Kamer JC. 1953 Excitement darkening and the effects of adrenaline on the melanophores of *Xenopus laevis*. Acta Endocrinol. **4**, 72-82. (10.1530/acta.0.0140072) 13104061

[27] Hjemdahl P, Linde P. 1983 Influence of circulating NE and Epi on adipose tissue vascular resistance and lipolysis in humans. Heart Circ. Physiol. **245**, 447-452. (10.1152/ajpheart.1983.245.3.H447) 6614192

[28] Tetens V, Lykkeboe G. 1988 Potency of adrenaline and noradrenaline for beta-adrenergic proton extrusion from red cells of rainbow trout, *Salmo gairdneri*. J. Exp. Biol. **134**, 267-280. (10.1242/jeb.134.1.267) 2833555

[29] Fabbri E, Capuzzo A, Moon TW. 1998 The role of circulating catecholamines in the regulation of fish metabolism: an overview. Comp. Biochem. Physiol. C **120**, 177-192. (10.1016/S0742-8413(98)10017-8) 9827031

[30] Browne RK, Seratt J, Vance C, Kouba A. 2006 Hormonal priming, induction of ovulation and *in-vitro* fertilization of the endangered Wyoming toad (*Bufo baxteri*). Reprod. Biol. Endocrinol. **4**, 1-11. (10.1186/1477-7827-4-34) 16790071 PMC1524778

[31] Silla AJ, Roberts JD. 2012 Investigating patterns in the spermiation response of eight Australian frogs administered human chorionic gonadotropin (hCG) and luteinizing hormone-releasing hormone (LHRHa). Gen. Comp. Endocrinol. **179**, 128-136. (10.1016/j.ygcen.2012.08.009) 22909973

[32] Rollins-Smith L, Reinert LK, Miera V, Conlon JM. 2012 Antimicrobial peptide defenses of the Tarahumara frog, *Rana tarahumarae*. Biochem. Biophys. Res. Commun. **297**, 361-367. (10.1016/S0006-291X(02)02217-9) 12237127

[33] Rollins-Smith L, Woodhams DC, Reinert LK, Vredenburg VT, Briggs CJ, Nielsen PF, Conlon JM. 2006 Antimicrobial peptide defenses of the mountain yellow-legged frog (*Rana muscosa*). Dev. Comp. Immunol. **30**, 831-842. (10.1016/j.dci.2005.10.005) 16330099

[34] Gomez D. 2006 AVICOL, a program to analyse spectrometric data. See http://sites.google.com/site/avicolprogram.

[35] Stückler S, Fuxjager MJ, Preininger D. 2022 Data from: Evidence that catecholaminergic systems mediate dynamic colour change during explosive breeding events in toads. Dryad Digital Repository. (10.5061/dryad.dbrv15f4b) PMC9580614 36259941

[36] Maia R, Eliason CM, Bitton PP, Doucet SM, Shawkey MD. 2013 pavo: An R package for the analysis, visualization and organization of spectral data. Methods Ecol. Evol. **4**, 906-913. (10.1111/2041-210X.12069)

[37] Rehberg-Besler N, Mennill DJ, Doucet SM. 2015 Dynamic sexual dichromatism produces a sex signal in an explosively breeding Neotropical toad: a model presentation experiment. Behav. Process. **121**, 74-79. (10.1016/j.beproc.2015.09.013) 26454154

[38] Reid SG, Bernier NJ, Perry SF. 1998 The adrenergic stress response in fish: control of catecholamine storage and release. Comp. Biochem. Physiol. C **120**, 1-27. (10.1016/S0742-8413(98)00037-1) 9827012

[39] Schreck CB. 1990 Physiological, behavioral, and performance indicators of stress. Am. Fish Soc. Symp. **8**, 29-37.

[40] Perry SF, Capaldo A. 2011 The autonomic nervous system and chromaffin tissue: neuroendocrine regulation of catecholamine secretion in non-mammalian vertebrates. Auton. Neurosci. **165**, 54-66. (10.1016/j.autneu.2010.04.006) 20547474

[41] Donner K, Yovanovich CAM. 2020 A frog's eye view: foundational revelations and future promises. Semin. Cell Dev. Biol. **106**, 72-85. (10.1016/j.semcdb.2020.05.011) 32466970

[42] Yovanovich CA, Koskela SM, Nevala N, Kondrashev SL, Kelber A, Donner K. 2017 The dual rod system of amphibians supports colour discrimination at the absolute visual threshold. Phil. Trans. R. Soc. B. **372**, 20160066. (10.1098/rstb.2016.0066) 28193811 PMC5312016

[43] Ali S, Naaz I. 2014 Comparative light and electron microscopic studies of dorsal skin melanophores of Indian toad, *Bufo melanostictus*. J. Microsc. Ultrastruct. **2**, 230-235. (10.1016/j.jmau.2014.07.002)

[44] Tang Z-J, Lue S-I, Tsai M-J, Yu T-L, Thiyagarajan V, Lee C-H, Huang W-T, Weng C-F. 2014 The hormonal regulation of color changes in the sexually dichromatic frog *Buergeria robusta*. Physiol. Biochem. Zool. **87**, 397-410. (10.1086/675678) 24769704

[45] Van Hove MA. 2021 Everyday physics: colors, light and optical illusions. Singapore: World Scientific Publishing Co Pte Ltd.

[46] Gardner KM, Mennill DJ, Newman AEM, Doucet SM. 2020 Social and physiological drivers of rapid colour change in a tropical toad. Gen. Comp. Endocrinol. **285**, 113292. (10.1016/j.ygcen.2019.113292) 31580882

[47] Camargo CR, Visconti MA, Castrucci AML. 1999 Physiological color change in the bullfrog, *Rana catesbeiana*. J. Exp. Zool. **283**, 160-169. (10.1002/(SICI)1097-010X(19990201)283:2<160::AID-JEZ6>3.0.CO;2-T) 9919686

[48] Filadelfi AM, Castrucci AM. 1994 Melatonin desensitizing effects on the *in vitro* responses to MCH, alpha-MSH, isoproterenol and melatonin in pigment cells of a fish (*S. marmoratus*), a toad (*B. ictericus*), a frog (*R. pipiens*), and a lizard (*A. carolinensis*), exposed to varying photoperiodic regimens. Comp. Biochem. Physiol. A **109**, 1027-1037. (10.1016/0300-9629(94)90252-6) 7828022

[49] Himes PJ, Hadley ME. 1971 *In vitro* effects of steroid hormones on frog melanophores. J. Investig. Dermatol. **57**, 337-342. (10.1111/1523-1747.ep12292565) 5315916

[50] Moore IT, Jessop TS. 2003 Stress, reproduction, and adrenocortical modulation in amphibians and reptiles. Horm. Behav. **43**, 39-47. (10.1016/s0018-506x(02)00038-7) 12614633

[51] Stückler S, Fuxjager MJ, Preininger D. 2022 Data from: Evidence that catecholaminergic systems mediate dynamic colour change during explosive breeding events in toads. Dryad Digital Repository. (10.5061/dryad.dbrv15f4b) PMC9580614 36259941

[52] Stückler S, Fuxjager MJ, Preininger D. 2022 Data from: Evidence that catecholaminergic systems mediate dynamic colour change during explosive breeding events in toads. Figshare. (10.6084/m9.figshare.c.6238491) PMC9580614 36259941

